# Trend of skin cancer mortality and years of life lost in China from 2013 to 2021

**DOI:** 10.3389/fpubh.2025.1522790

**Published:** 2025-02-12

**Authors:** Jingyi Li, Jiaqi Zeng, Yuanchao Yang, Biran Huang

**Affiliations:** ^1^Department of Dermatology, Huizhou First Hospital, Huizhou, China; ^2^Department of Dermatology, Zhuhai People's Hospital (Zhuhai Clinical Medical College of Jinan University), Zhuhai, China

**Keywords:** China, disease burden, skin cancer, mortality rate, years of life lost

## Abstract

**Background:**

The mortality rates of skin cancer in Chinese population are increasing. However, research on skin cancer trends in China is limited. This study aimed to estimate the mortality trends of skin cancer in China within 2013–2021.

**Methods:**

A retrospective analysis of skin cancer deaths within 2013–2021 was performed using the China death cause surveillance dataset compiled by the National Health Commission Statistics Information Center and the China Center for Disease Control and Prevention Chronic Non-Communicable Diseases Prevention and Control Center. The mortality rates of skin cancer were stratified by gender, age group, and area (urban or rural).

**Results:**

From 2013 to 2021 in China, the crude mortality rate (CMR) of skin cancer increased, and the age-standardized mortality rate (ASMR), and age-standardized years of life lost (YLL) rate decreased. The ASMR and age-standardized YLL rate were 0.85/100,000 and 18.95/100,000 in 2013, respectively, and decreased to 0.75/100,000 and 16.84/100,000, respectively, in 2021. From 2013 to 2021, the CMR, ASMR, and age-standardized YLL rate of skin cancer were higher in males than in females and higher in rural areas rather than in urban ones. In terms of the highest age-specific mortality rate, it appeared in the age group of over 85 years old.

**Conclusion:**

The burden of skin cancer remained heavily from 2013 to 2021 in China. Especially males, older adult, and rural residents had higher mortality. Thus, effective measures and strategies should be taken to reduce the incidence and mortality of skin cancer.

## Introduction

Skin cancer is one of the most common carcinomas worldwide, affecting all races and socioeconomic groups in all geographic areas (1). Based on cellular origin, these diseases are primarily categorized as melanoma (MM) and non-melanoma skin cancer (NMSC), and NMSC is divided into basal cell carcinomas (BCCs) and squamous cell carcinomas (SCCs) as the major histologic subtypes (2). Skin cancer incidence is rising worldwide, covering all age groups especially middle-aged and older adult people. According to the International Agency for Research on Cancer, 1.518 million skin cancer new cases were diagnosed, with 121,000 new deaths worldwide in 2020 (3). According to Skin Cancer Foundation Statistics, one in every three cancers diagnosed is skin cancer (4, 5). Skin cancer burdens vary widely across countries, and higher numbers, incidences, and mortality of skin cancer are observed in high sociodemographic index regions (1). New Zealand and Australia have the highest incidence rates of MM in the world (50 and 48 cases per 100,000 persons, respectively), followed by the USA and Europe (6). In terms of BCC, Australia, Europe, and USA have the highest incidence rates (6). The incidence of skin cancer in China is lower than that in European countries and America. However, the incidence of MM has increased over the past two decades in China, reaching 0.62 per 100,000 in 2022 as estimated, and the mortality rate was 0.38 per 100,000 (7). The incidence and burden of skin cancer are associated with demographic trends, socioeconomic development, race, and risk exposures (1). Exposure to ultraviolet radiation (UVR) is the most well-established risk factor for skin cancer (5).

Mortality rates have been used to characterize people’s health status, however, those measures do not fully account for the burden of premature mortality (8). Compared with mortality, years of life lost (YLL) is an important and common disease burden indicator, which takes the death count and life expectancy at death into consideration (9). At present, researchers primarily focus on other cancers in China. Although the incidence and mortality of MM in China have been studied (10, 11), publications on the burden of skin cancer are few. Accordingly, the current work aimed to assess the disease burden of skin cancer in China and to provide policy recommendations regarding interventions for reducing the burden of skin cancer.

## Materials and methods

### Data sources

Skin cancer mortality data in our study were obtained from the China death cause surveillance dataset^1^ for the period of 2013–2021. These data were acquired from the national mortality surveillance system with 605 surveillance points across China and about 24% of the Chinese population covered. They were compiled by the National Health Commission Statistics Information Center and the China Center for Disease Control and Prevention Chronic Non-Communicable Diseases Prevention and Control Center. Skin cancer mortality and the number of deaths were calculated by gender, age groups, and areas (urban or rural). Cause-specific mortality and deaths data for skin cancer were collected under strict quality-control measures with good national and provincial representation. We used the China’s seventh census in 2020 as the standard population to estimate the age-standardized rate, which were obtained from the China National Bureau of Statistics.

### Years of life lost estimation

YLL is a measure of premature death. It is calculated as the sum of each death multiplied by the standard life expectancy at each age. That is *YLL (c, s, a, t)* = *N (c, s, a, t) × L (s, a)* where: *c*, cause; *a*, age; *s*, sex and *t*, year; *N (c, s, a, t)* is the number of deaths due to the cause for the given age and sex in year; *L (s, a)* is a standard loss function specifying years of life lost for a death at age for sex (11). We used a theoretical minimal risk reference life table to calculate YLL of skin cancer from 2013 to 2021.

### Statistical analysis

The number of deaths, crude mortality rate (CMR), YLL, age-standardized mortality rate (ASMR), and age-standardized YLL rate caused by skin cancer from 2013 to 2021 were analyzed by sex, age groups, and areas (urban and rural). All statistical analyses were performed using SPSS 20.0 (*p* < 0.05 indicates statistical significance) and graphed with Origin 2021.

## Results

### Mortality rate and disease burden of skin cancer

**Figure 1:**
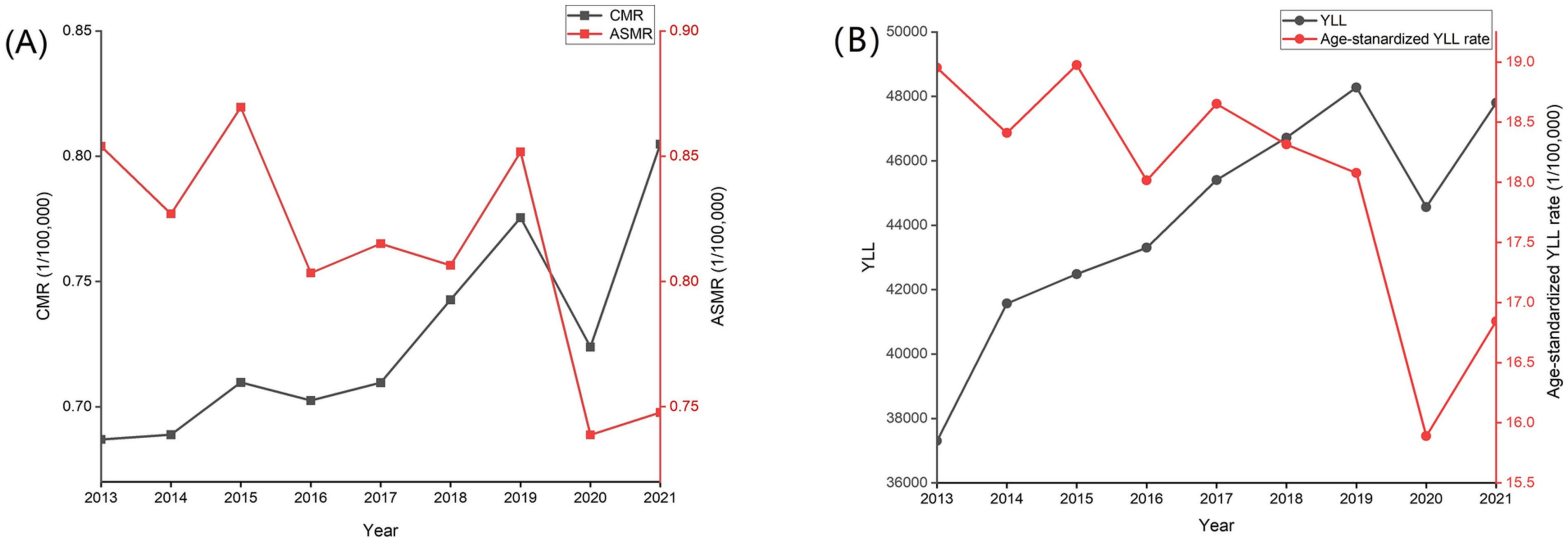
Trends in mortality rate, age-standardized mortality rate **(A)**, age-standardized YLL rate and YLL **(B)** of skin cancer in China, 2013–2021.

From 2013 to 2021, skin cancer accounted for 17,261 deaths (9,495 males, 7,766 females) and 397421.34 YLL (227974.64 males and 169446.68 females) in China. The overall CMR of skin cancer, which tended to increase in the national surveillance areas, was between 0.69/100000 and 0.80/100000 from 2013 to 2021 (trend chi-square value = 33.06, *p* < 0.01). Between 2013 and 2021, the ASMR of skin cancer decreased from 0.85/100,000 to 0.75/100,000 (trend chi-square value = 150.85, *p* < 0.01). The age-standardized YLL rate of skin cancer showed similar trend to the ASMR from 2013 to 2021 and decreased from 18.95/100,000 to 16.84/100,000 (Figure 1).

### Age- and sex-specific mortality rates and disease burden of skin cancer

**Figure 2:**
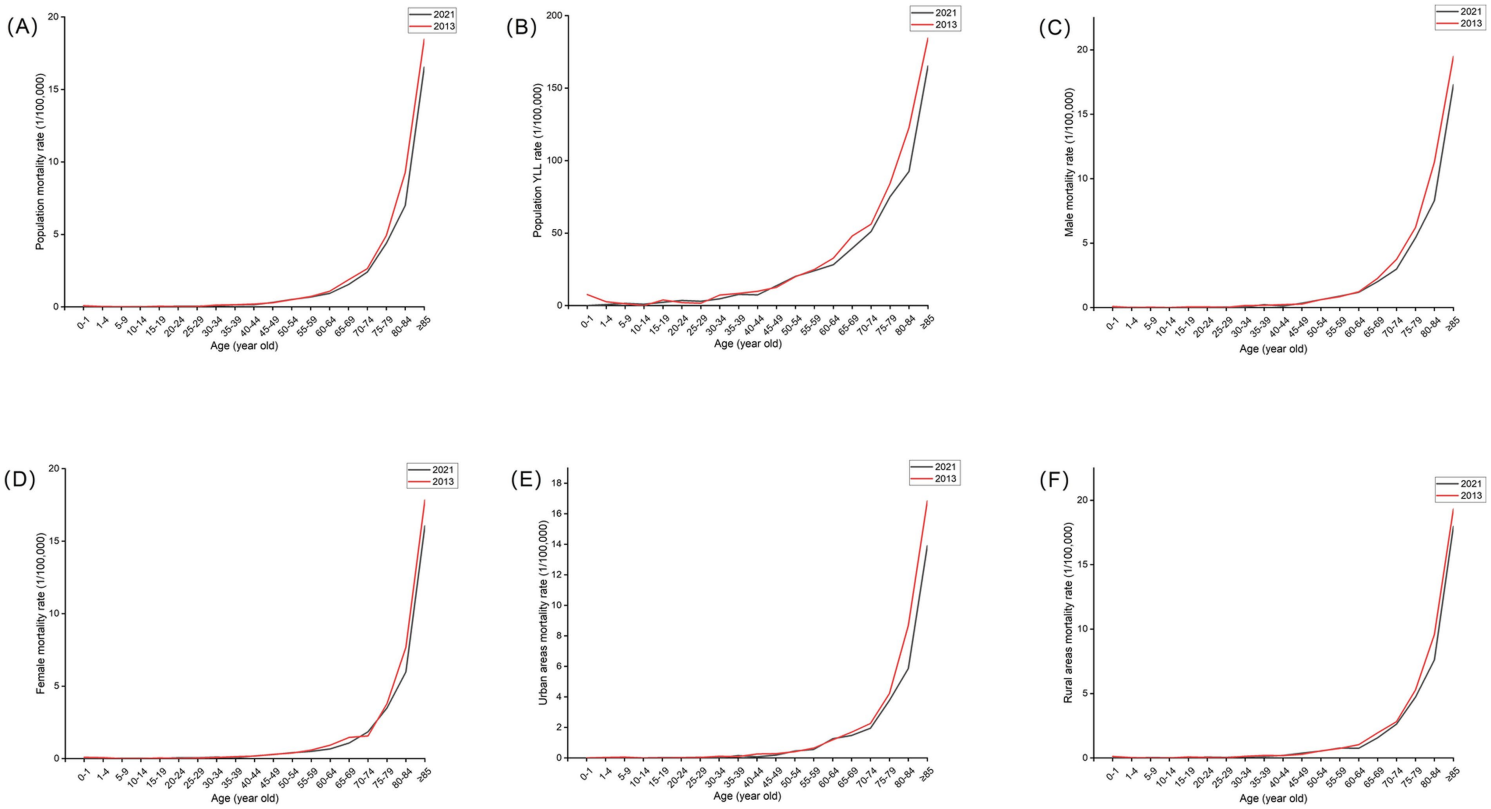
Age-specific mortality rates and YLL rate of skin cancer in whole population **(A,B)**, age-specific mortality rates by sex **(C,D)** and region **(E,F)** for skin cancers in China in 2013 and 2021.

The CMR of skin cancer generally increased with age from 2013 to 2021. It increased from 45 years old and peaked at aged over 85 years. YLL rate showed similar trend to the CMR. We analyzed the age-specific mortality rates of skin cancer for different years. Compared with 2013, the CMR of skin cancer in most age groups decreased in 2021 in male, female, both sexes, and rural and urban areas (Figure 2).

**Figure 3:**
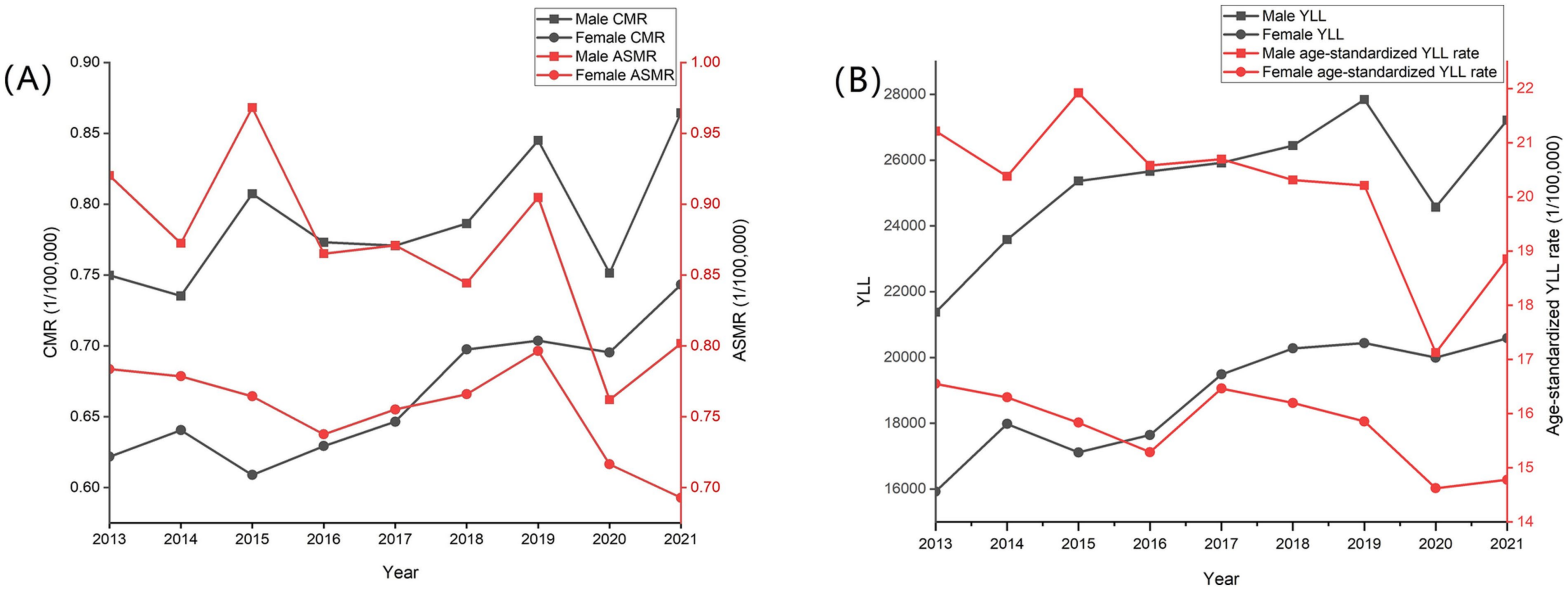
Trends in mortality rate, age-standardized mortality rate **(A)**, age-standardized YLL rate and YLL **(B)** of skin cancer by sex in China, 2013–2021.

In terms of sex, the CMR, ASMR, and age-standardized YLL rate of males were consistently higher than that of females from 2013 to 2021 (*p* < 0.01). In males and females, the CMR of skin cancer tended to increase (trend chi-square value = 9.64 and 26.60, respectively, *p* < 0.01), and the ASMR decreased (trend chi-square value = 125.91 and 31.76, respectively, *p* < 0.01). Compared with 2013, the ASMR of skin cancer for males and females decreased by 12.88 and 11.59% respectively, and the age-standardized YLL rate for males and females decreased by 11.11 and 10.71%, respectively, in 2021 in China (Table 1; Figure 3).

**Table 1:** The sex-specific mortality rate, age-standardized mortality rate, and disease burden of skin cancer from 2013 to 2021.

Year	Male					Female				
	Death numbers	CMR	ASMR	YLL	Age-standardized YLL rate	Death numbers	CMR	ASMR	YLL	Age-standardized YLL rate
2013	868	0.75	0.92	21375.48	21.22	693	0.62	0.78	15926.17	16.55
2014	951	0.74	0.87	23586.65	20.38	796	0.64	0.78	17981.97	16.30
2015	1,056	0.81	0.97	25366.07	21.92	772	0.61	0.76	17115.49	15.83
2016	1,042	0.77	0.87	25657.66	20.58	818	0.63	0.74	17645.04	15.29
2017	1,062	0.77	0.87	25918.31	20.70	862	0.65	0.76	19486.06	16.46
2018	1,089	0.79	0.84	26442.36	20.31	933	0.70	0.77	20274.61	16.20
2019	1,188	0.85	0.90	27842.30	20.21	959	0.70	0.80	20437.09	15.85
2020	1,063	0.75	0.76	24571.27	17.13	953	0.70	0.72	19994.58	14.62
2021	1,176	0.86	0.80	27214.54	18.86	980	0.74	0.69	20585.67	14.78

CMR, ASMR and age-standardized YLL rate (1/100,000). CMR, crude mortality rate; ASMR, age-standardized mortality rate; YLL, years of life lost.

### Regional differences in China

**Figure 4:**
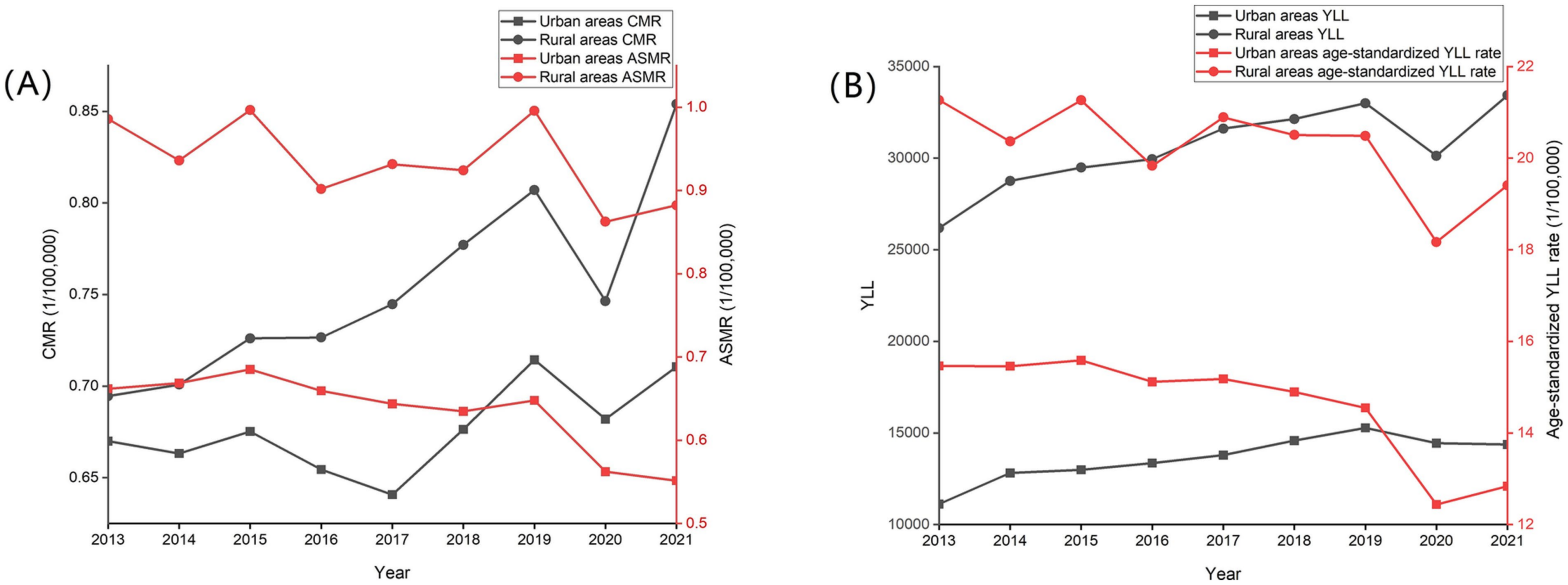
Trends in mortality rate, age-standardized mortality rate **(A)**, age-standardized YLL rate and YLL **(B)** of skin cancer by region in China, 2013–2021.

In general, the CMR, ASMR, and age-standardized YLL rate in urban areas were lower than that in rural areas within 2013 to 2021 (*p* < 0.01). CMR increased in urban and rural areas but was not statistically significant in urban areas, whereas ASMR decreased (trend chi-square value = 111.55 and 56.13, respectively, *p* < 0.01). Compared with 2013, the ASMR of skin cancer in rural and urban areas decreased by 16.68 and 10.51% in 2021, respectively. Age-standardized YLL rate also decreased in 2021 compared with 2013, whereas the rates in rural and urban areas decreased by 16.99 and 8.75%, respectively (Table 2; Figure 4).

**Table 2:** Regional differences of mortality rate, age-standardized mortality rate, and disease burden of skin cancer from 2013 to 2021.

Year	Urban areas					Rural areas				
	Death numbers	CMR	ASMR	YLL	Age-standardized YLL rate	Death numbers	CMR	ASMR	YLL	Age-standardized YLL rate
2013	471	0.67	0.66	11118.09	15.46	1,090	0.69	0.99	26183.56	21.27
2014	537	0.66	0.67	12810.17	15.46	1,210	0.70	0.94	28758.45	20.37
2015	560	0.68	0.69	12992.63	15.59	1,268	0.73	1.00	29488.94	21.27
2016	579	0.65	0.66	13358.23	15.12	1,281	0.73	0.90	29944.47	19.84
2017	587	0.64	0.64	13787.41	15.18	1,337	0.74	0.93	31616.97	20.89
2018	629	0.68	0.63	14581.23	14.90	1,393	0.78	0.92	32135.74	20.51
2019	675	0.71	0.65	15276.39	14.55	1,472	0.81	1.00	33003.00	20.49
2020	666	0.68	0.56	14437.85	12.44	1,350	0.75	0.86	30128.00	18.17
2021	652	0.71	0.55	14368.92	12.84	1,504	0.85	0.88	33431.29	19.41

CMR, ASMR and age-standardized YLL rate (1/100,000). CMR, crude mortality rate; ASMR, age-standardized mortality rate; YLL, years of life lost.

## Discussion

Skin cancer is a serious malignant tumor worldwide and an important public-health problem. Numerous risk factors for skin cancer exist, including long-time ultraviolet (UV) exposure, age, gender and genetic susceptibility (6). The most common skin cancers are MM, SCC, and BCC. In 2019, the estimated worldwide skin cancer incidences were 3.56/100,000 for MM, 30.3/100,000 for SCC, and 48.80/100,000 for BCC, whereas the mortality were 1.63/100,000 for MM, 0.73/100,000 for SCC (1). Skin cancer incidences and mortality rate vary greatly among different countries and regions. Studies have shown that the incidence rate of skin cancer was 398.12 per 100,000 in England in 2019 (12) and 7.4 per 100,000 person-years in Singapore within 2003–2006 (13). Meanwhile, the overall prevalence of skin cancer was 0.17–0.45 per 100,000 within 2004–2015 in South Korea (14). In Iran, the mortality rate of skin cancer was approximately 0.5 per 100,000 from 2006 to 2010 (15). In China, mortality and YLL rate of skin cancer in 2020 was 0.72 and 12.37 per 100,000, respectively (16). Compared with previous studies in China, the CMR, ASMR, and age-standardized YLL rate of skin cancers in this study increased to 0.80/100,000, 0.75/100,000, and 16.84/100,000 in 2021, respectively.

Solar UVR is classified as a carcinogen by the World Health Organization (17). Studies have shown that a higher skin cancer morbidity and mortality in outdoor workers and pilots probably because they are exposed to higher levels of UV radiation in outdoor works and ionizing radiation in pilots (17–19). Moreover, MM increases as the climate changes and the ozone layer thins. Studies show that ozone-layer depletion increases the amount of harmful solar UV radiation reaching the Earth, resulting in increased skin cancer incidence (4, 5). For every 10% decrease in ozone, MM and new non-melanoma skin cancers are estimated to increase to 4,500 MM and 300,000, respectively (4, 5).

Apart from UVR, demographic and sociological factors are also important factors in the increased incidence of skin cancer, such as increased overall life expectancy, sunbathing and lack of sunscreen use (20). Concerns related to sun-protection attitudes and behaviors differ markedly across gender, age, income, and education groups. Women, younger, and people with high socioeconomic status have more correct sun-protection beliefs (21). In our study, we found differences in skin cancer mortality according to gender, with higher rates in males than in females. This is similar to Zhang et al.’s results, i.e., males have higher incidence and mortality rates of skin cancer than females on a global scale (1). The reason may be that men usually have less sun-protection behavior and more UVR exposure. Many men have expressed using umbrella and sunscreen as feminine activities (22). Additionally, males have longer daily sunlight exposure time than females from 07: 00 to 17: 00, especially from 10:00 to 14:00 (21). Advanced age is an important risk factor for skin cancer, and older age is associated with a higher risk of skin cancer (1, 20). Perhaps older people may have higher cumulative UVR exposure. Globally, skin cancer carries a high burden, especially among the older adult population, and it increases from the age of 55 years (1). Similar to previous studies, we found that skin cancer mortality increased with age starting at age 45 years and was the highest in individuals over 85 years old. In line with previous results (23), we also found a higher skin cancer mortality in rural areas than in urban ones. The reason may be that individuals living in rural areas are exposed to UV light for a longer time while working in the field, resulting in a higher cumulative dose of UVR and thus skin cancer incidence and mortality. Moreover, compared with people living in urban areas, shade seeking and sunscreen use are less frequent in the rural counterparts (24). Thus, healthy lifestyle to reduce UV radiation exposure is the key to prevent the occurrence of skin cancer. Nevertheless, many Chinese people have a large deficit in knowledge and attitude regarding UV radiation (21). Only 55.2% of the participants are found to be aware that UV radiation causes skin cancer and almost 80% of participants never use sunscreen during sun exposure, especially males (21). Hence, to reduce skin cancer morbidity and mortality in China, more efforts and measures should be made to improve people’s knowledge about skin cancer prevention and UVB radiation protection, especially males and rural residents.

From 2013 to 2021, we observed skin cancer ASMR of 0.75 per 100,000 in 2020, which was lower compared to the value before the pandemic such as 2019 (χ^2^ = 113.17, *p* < 0.01). The reason may be that patients were reluctant to seek medical care for skin cancer during the COVID-19 pandemic. As a result, mortality generally fell according to statistics from the China death cause surveillance dataset. Similarly, the number of newly diagnosed skin cancers in England declined during the COVID-19 pandemic (25).

In our study, we found that the ASMR and age-standardized YLL rate continue to decreased from 2013 to 2021. However, skin cancer deaths continue to increase, and the burden of skin cancer remains heavily and should be highly valued. This can be a consequence of UVR and the aging of the population. Strategies should be developed to reduce the incidence and mortality of skin cancer, and the focus should be on preventive measures, such as UV protection, public education, and self-exams, particularly in males, older adult, and rural residents.

However, this study had several limitations. Firstly, the available datas were limited, preventing stratification of disease burden for various types of skin cancer. Secondly, in the absence of occupation and sun protection habits data, the association between skin cancer burden and UVR exposure is speculative. Although we failed to obtain the above details from existing databases within a short time frame, we still strive to collect relevant data and analyze the disease burden for different types of skin cancer and the relationship between skin cancer and UVR exposure to optimize future prevention and control policies.

## Data Availability

Publicly available datasets were analyzed in this study. This data can be found here: Skin cancer mortality data in our study were obtained from the China death cause surveillance dataset for the period of 2013–2021 (https://ncncd.chinacdc.cn/jcysj/siyinjcx/syfxbg/). They were compiled by the National Health Commission Statistics Information Center and the China Center for Disease Control and Prevention Chronic Non-Communicable Diseases Prevention and Control Center.

## Footnotes

^1^ https://ncncd.chinacdc.cn/jcysj/siyinjcx/syfxbg/
